# Engineering a Pseudo-26-kDa *Schistosoma* Glutathione Transferase from *bovis*/*haematobium* for Structure, Kinetics, and Ligandin Studies

**DOI:** 10.3390/biom11121844

**Published:** 2021-12-07

**Authors:** Neo Padi, Blessing Oluebube Akumadu, Olga Faerch, Chinyere Aloke, Vanessa Meyer, Ikechukwu Achilonu

**Affiliations:** 1 Protein Structure-Function and Research Unit, School of Molecular and Cell Biology, Faculty of Science, University of the Witwatersrand, Braamfontein, Johannesburg 2050, South Africa; 1482699@students.wits.ac.za (N.P.); 1497819@students.wits.ac.za (B.O.A.); olga.faerch@wits.ac.za (O.F.); chinyere.aloke@wits.ac.za (C.A.); 2 Functional Genomics and Immunogenetics Laboratory, School of Molecular and Cell Biology, Faculty of Science, University of the Witwatersrand, Braamfontein, Johannesburg 2050, South Africa; vanessa.meyer@wits.ac.za

**Keywords:** *Schistosoma*, glutathione transferase, praziquantel, CDNB assay, kinetics, isothermal titration calorimetry, thermal shift assay, spectroscopy, inhibition, bromosulfophthalein

## Abstract

Glutathione transferases (GSTs) are the main detoxification enzymes in schistosomes. These parasitic enzymes tend to be upregulated during drug treatment, with *Schistosoma haematobium* being one of the species that mainly affect humans. There is a lack of complete sequence information on the closely related *bovis* and *haematobium* 26-kDa GST isoforms in any database. Consequently, we engineered a pseudo-26-kDa *S. bovis*/*haematobium* GST (Sbh26GST) to understand structure–function relations and ligandin activity towards selected potential ligands. Sbh26GST was overexpressed in *Escherichia coli* as an MBP-fusion protein, purified to homogeneity and catalyzed 1-chloro-2,4-dinitrobenzene-glutathione (CDNB-GSH) conjugation activity, with a specific activity of 13 μmol/min/mg. This activity decreased by ~95% in the presence of bromosulfophthalein (BSP), which showed an IC_50_ of 27 µM. Additionally, enzyme kinetics revealed that BSP acts as a non-competitive inhibitor relative to GSH. Spectroscopic studies affirmed that Sbh26GST adopts the canonical GST structure, which is predominantly α-helical. Further extrinsic 8-anilino-1-naphthalenesulfonate (ANS) spectroscopy illustrated that BSP, praziquantel (PZQ), and artemisinin (ART) might preferentially bind at the dimer interface or in proximity to the hydrophobic substrate-binding site of the enzyme. The Sbh26GST-BSP interaction is both enthalpically and entropically driven, with a stoichiometry of one BSP molecule per Sbh26GST dimer. Enzyme stability appeared enhanced in the presence of BSP and GSH. Induced fit ligand docking affirmed the spectroscopic, thermodynamic, and molecular modelling results. In conclusion, BSP is a potent inhibitor of Sbh26GST and could potentially be rationalized as a treatment for schistosomiasis.

## 1. Introduction

Schistosomiasis is a waterborne disease caused by a chronic infection from a parasitic blood fluke of *Schistosoma*. It is one of the most neglected tropical diseases, second to malaria [1]. This disease affects both humans and animals, thus posing a medical and veterinary concern that drags the socio-economic state of affected regions. According to conservative estimates, over 230 million people worldwide are infected with schistosomiasis, dominating sub/tropical regions such as sub-Saharan Africa [2,3]. The infection induces a range of morbidities ranging from fever to fibrotic organ damage that culminates in death. Since 1982, the only available drug for treating schistosomiasis has been the anti-helminthic praziquantel (PZQ). However, PZQ is only effective against the host antibody response caused by egg-producing adult schistosome worms and not against the poorly detected juvenile schistosomes [4]. The exact mode of molecular targeting by PZQ is also still not fully understood, and mass administration of the drug is associated with the threat of drug resistance [5]. These factors emphasize the need for a rational-based approach towards discovering a new generation of anti-helminthics. Therefore, medications should be developed by analyzing the structure and function of a target molecule rather than using arbitrary drugs in the hope of eliciting a therapeutic effect.

Due to the lack of phase 1 detoxification enzymes (e.g., cytochrome P450) in schistosomes, these parasites express two isoforms of glutathione transferases (GSTs) as a sole primary defence mechanism against toxins produced by internal physiological changes and the external host immune response [6]. Since GSTs play a vital role in detoxifying endogenous and exogenous electrophiles through the catalysis of glutathione (GSH) conjugation, these proteins tend to experience upregulation during drug treatment. In schistosomes, GSTs are highly expressed during the egg stage to aid with fecundity. Reports have shown that silencing the 28-kDa isoform compromises egg growth due to redox reaction imbalance and eventually decreases prolificacy [7,8]. Conversely, the 26-kDa isoform displays involvement in toxin storage and transportation. This includes minimization of lipid peroxidation, which causes oxidative damage [8], and heme built up during host erythrocyte metabolism that would otherwise form hematin and block the parasite’s gut [9,10]. These findings suggest the potential of GSTs as an attractive target for drugs and vaccines that may prevent re-infection.

*Schistosoma* species that primarily infect humans include the urinary *S. haematobium* and the intestinal *S. mansoni* and *S. japonicum*. *S. haematobium* was chosen as the subject of this study due to the significant impact of this parasite in Africa and the presence of unusual pathological characteristics, namely, hydronephrosis and bladder and urethral fibrosis, under chronic infections. The zoonotic character of this parasite further allows the natural interaction with livestock schistosomiasis, thereby facilitating the bidirectional introgressive hybridization with *S. bovis* [7]. This interaction supports the close relationship between the two species and an insignificant phylogenetic distance. What is alarming is that laboratory hybrids tend to depict heterosis (diverse intermediate host, faster maturation, and increased fecundity) [11], which might collapse the treatment of schistosomiasis. Unfortunately, studies on GSTs of these species have been limited by the lack of a complete open reading frame (ORF) encoding the 26-kDa isoforms in both *S. haematobium* and *bovis*. In particular, 12 residues are missing from the *N*-terminus Met despite the sequence of *S. bovis* being partially complete [12,13,14]. Our preliminary studies have shown that the sequence encoding the putative 26-kDa GST in *S. haematobium* and *bovis* appears to be 100% identical in both amino acid sequence and mRNA sequence (Figure S1).

While the 3D structure of the 26-kDa isoform of *S. bovis/haematobium* currently remains unelucidated, homologous species were used as a model for the classical GST structure. The structures show a globular homodimer with 218 amino acids that folds into two subunits. The *N*-terminus thioredoxin-like domain (βαβαββ) houses GSH (G-site), in proximity to the dimer interface. In contrast, an all α-helical C-terminus domain contains the electrophilic substrate site (H-site) [15]. The active site in each monomer is characterized by fewer polar residues in the H-site than in human cytosolic GSTs, thus rendering this site more hydrophobic with different substrate sensitivity. Furthermore, the dimer interface of the enzyme in schistosomes can bind non-substrates without affecting catalysis, as proven by the study of PZQ binding in a homolog of Sbh26GST from the *japonicum* species (Sj26GST), and this site is usually not implicated in human GSTs. These differences provide a structural basis for developing a new generation of anti-helminth drugs [16].

Due to the ligandin functions of GST, these proteins can also bind a variety of electrophiles. Some studies indicate that known inhibitors of human GSTs, such as ethacrynic acid, usually binds at the H-site. However, in schistosomes, some inhibitors, such as ellagic acid, bind both the H-site and dimer interface of Sj26GST [17], while others, such as BSP, favor binding the dimer interface of Sj26GST [18]. This knowledge provides an alternative site for non-substrate xenobiotics to regulate GSTs. The ligands of interest in this study, PZQ, bromosulfophthalein (BSP), and artemisinin (ART), display bulky rings with some hydrophobic character. BSP is an amphipathic anion dye initially used for liver functionality, whereby healthy patients show rapid sequestration from the blood through bile conjugates [19]. However, it is also used as a probe for catalytic properties of GSTs, since it alters the intracellular concentration of GSH [20,21]. ART is a sesquiterpene lactone derived from sweet wormwood to treat malaria, displaying an efficacy that can extend to phylogenetically unrelated parasites such as schistosomes [22]. Given that the mentioned drugs are well studied and already available in the market, repurposing these for the treatment of schistosomiasis will be shorter and less costly than developing novel drugs.

Since there is a need for rational drug discovery against schistosomes using GST as a target, it is essential to generate a complete picture of the biochemistry of both isoforms in all human *Schistosoma* parasites. Using available sequence information on the putative 26-kDa GST in *S. haematobium* and *bovis* and the complete sequence (ORF) from other species of the parasite, we constructed a pseudo Sbh26GST for recombinant expression and purification using the pMALc5X *E. coli* expression system. The structure, function, and ligandin properties of the enzyme were studied in the hope of adding to the body of knowledge on potential inhibitors that can be designed or repurposed as new-generation anti-helminthics.

## 2. Materials and Methods

### 2.1. Materials

All reagents and fine chemicals were of analytical grades. The vector encoding the pseudo-26-kDa GST from *S. bovis/haematobium* was synthesized and subcloned into a pMAL-c5X vector by GenScript Corporation (Piscataway, NJ, USA). *E. coli* T7 Express competent cells were purchased from New England Biolabs (Ipswich, MA, USA). Ampicillin, chloramphenicol, IPTG, GSH, CDNB, BSP, PZQ, and ART were from Sigma-Aldrich^®^ (St. Louise, MO, USA), whereas ANS was purchased from Sigma-Merck (South Africa). The 2D representations of the ligands are shown in Figure S2.

### 2.2. Vector Construction

**Figure 1:**
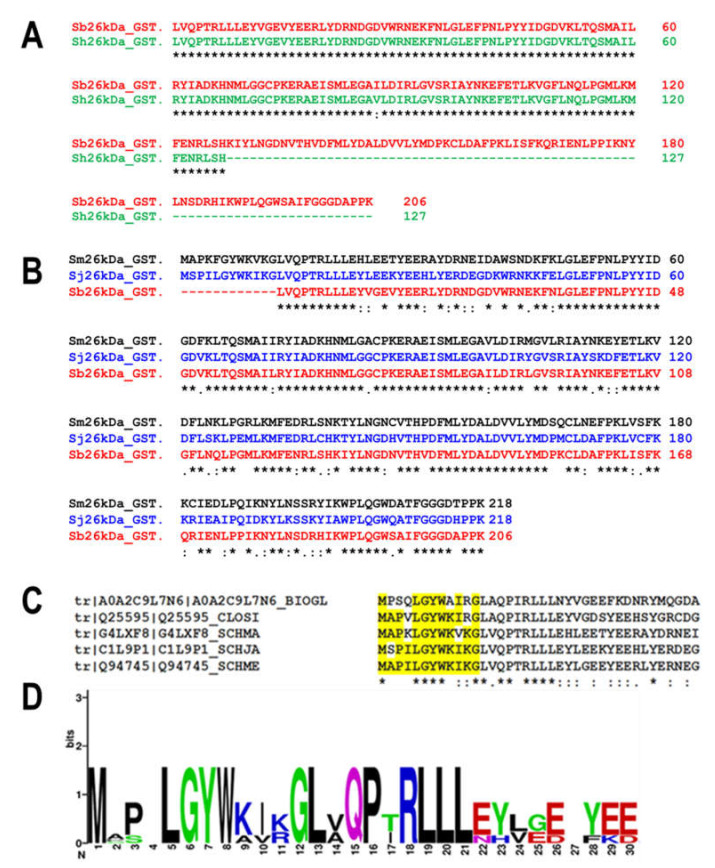
Sequence analysis aiding in the construction of the pseudo-*Schistosoma bovis/haematobium* 26-kDa GST. (**A**) Alignment of the fragments of the putative 26-kDa GST from *S. bovis* (**red**) and *S. haematobium* (**green**) using Clustal Omega. Excluding the additional C-terminus region in *bovis*, these sequences share 99.9% sequence identity. (**B**) Alignment of the putative 26-kDa *S. bovis* GST fragment (**red**) with the 26-kDa GST isoforms of *S. japonicum* (**blue**) and *S. mansoni* (**black**). This highlights the missing 12 amino acids in the *N*-terminus, confirming that the fragmented sequence is likely to be the 26-kDa isoform from *bovis* and/or *haematobium*. (**C**) Alignment of the identified five top-scoring sequences from BLAST analysis using dendrogram clustering (implemented in the Clustal Omega algorithm). (**D**) WebLogo analysis of the alignment in ***C*** indicating the consensus sequence to be MAP**A**LG**Y**WKIKG. Alanine is the 4th amino acid, and the catalytic Tyr (in **bold**) is the 7th. The UniProt Kb accession numbers for 26-kDa *S. bovis*, *S. haematobium*, *S. japonicum*, and *S. mansoni* GSTs are A0A430Q9J5, A0A6A5D1Y9, P08515, and P15964, respectively.

Several sequence analysis tools were used to construct the pseudo-26-kDa GST similar to native Sh26GST and Sb26GST, as shown in Figure 1. The partial polypeptide and nucleotide sequences for Sh26GST (UniProtKB ID: A0A6A5D1Y9) and Sb26GST (UniProtKB IDL: A0A430Q9J5) were isolated from the UniProtKB database. Clustal Omega was used to confirm that these sequences are related to the Sj26GST. The Clustal Omega analysis confirmed that partial sequences are the 26-kDa isoform of *Schistosoma* GST and not the 28-kDa isoform. However, the first 12 amino acids are missing in both sequences. This was followed by Clustal Omega analysis of the nucleotide sequences from both *bovis* and *haematobium*. The analysis showed 99.9% sequence identity confirming that both sequences could have stemmed from a species resembling *bovis* and *haematobium*. Since the originating species appears inconsequential, this pseudo-GST will herein be referred to as *S. bovis-haematobium* 26-kDa GST (Sbh26GST). To complete the 12 missing amino acids, BLAST was used to identify evolutionarily related sequences in the database. After that, top-scoring sequences were subjected to multiple sequence analysis to identify a consensus first 12 amino acids sequence. The potential ORF was derived by merging the supposed nucleotide sequence encoding the first 12 amino acids with the nucleotide sequence encoding the fragmented *S. bovis* 26-kDa GST (EMBL ID: RTG84353). The ORF was also engineered to encode a thrombin-cleavable hexahistidine tag. The final sequence was codon-optimized for expression in *E. coli* and cloned into a pMAL-c5X for expression as a maltose-binding protein-fusion protein. The optimization, synthesis, and subcloning were performed by GenScript Corporation (Piscataway, NJ, USA).

### 2.3. Protein Over-Expression

T7 *E. coli* competent cells were transformed with the pMAL-c5X vector encoding Sbh26GST. Single colonies were cultured overnight in 2YT media supplemented with 50 µg/mL of ampicillin and 35 µg/mL of chloramphenicol at 37 °C, 250 rpm. The overnight cultures were diluted 1/50 in fresh 2YT media and incubated (37 °C, 250 rpm) until an OD_600_ ~0.5 was reached. The cultures were then incubated on ice for 10 min before induction with 0.5 mM IPTG for 6 h at 30 °C, 250 rpm. The cells were harvested by centrifugation at 5000× *g,* 4 °C for 15 min. The pellet collected was ~4 g of wet cells per liter of culture and was resuspended in 50 mL of buffer A (50 mM Tris-HCl, pH 7.5, and 0.02% (*w*/*v*) NaN_3_) before storing at −80 °C.

### 2.4. Affinity Chromatography Purification

The frozen cell suspension was thawed at room temperature, after which the cellular components of the cells were released by sonication on ice. The soluble cell fraction was collected in the supernatant by centrifugation at 18,000× *g*, 4 °C for 20 min. The supernatant was subjected to maltose-binding protein (MBP)-amylose resin pre-equilibrated with buffer A. The column was washed with buffer A containing 0.05% (*v*/*v*) Tween-20 to eliminate unbound protein, and elution was conducted with 10 mM maltose. The fractions with eluted protein were pooled and dialyzed overnight against 50 mM Tris-HCl, pH 7.5, 5 mM CaCl_2_, and 0.02% (*w*/*v*) NaN_3_ (buffer B) at 4 °C with two changes of ~500× sample size. The protein was treated overnight with thrombin (0.5 U/mg of recombinant protein) at room temperature. The cleaved protein was subjected to immobilized metal affinity chromatography (IMAC), using immobilized nickel chelate resin pre-equilibrated with buffer B. The column was washed with buffer B to remove any protein impurities, and bound protein was eluted with 50 mM Tris-HCl, pH 7.5, 500 mM NaCl, 300 mM imidazole, and 0.02% (*w*/*v*) NaN_3_. Finally, fractions with the desired protein were pooled and dialyzed overnight against buffer A at 4 °C with two buffer changes of 200x sample size. The purity was analyzed qualitatively by reducing SDS–PAGE [23] and quantitatively by spectrophotometry using the Beer–Lambert law and a theoretically estimated molar extinction coefficient of 79,760 M^−1^·cm^−1^.

### 2.5. GSH–CDNB Conjugation Assay

The catalytic activity of Sbh26GST at varying enzyme concentrations was monitored, in both the presence and absence of 0.5 mM PZQ, ART, and BSP, through the reduced GSH–CDNB conjugation assay. During this assay, dinitrophenyl thioether chromophore, 1-(*S*-glutathionyl)-2,4 dinitrobenzene (ε_340_ = 9 600 M^−1^·cm^−1^) is produced which absorbs at 340 nm [24]. The reaction buffer contained 100 mM sodium phosphate, 1 mM EDTA, 5 mM DTT, 0.02% NaN_3_, 5 mM GSH, 1 mM CDNB (3% (*v*/*v*) ethanol); pH 6.5. Reactions were carried out in triplicate on a Jasco V-730 BIO spectrophotometer at 20 °C for 60 s.

The linear progress curves were corrected for background non-enzymatic reaction rates. The assay was also used for half-maximal inhibitory (IC_50_) determination of the top inhibitor, BSP. Varying ligand concentrations were added to 100 nM of Sbh26GST and incubated for 30 min at 20 °C, after which CDNB was added to initiate the reaction. A non-linear four-parameter regression curve was fitted to the data:

$$y=\frac{bottom+\left(top-bottom\right)}{1+10^{\left(({\log\;\mathrm{IC}}_{50}-x)\times hillslope\right)}}$$

where *y* is the enzyme activity (%), *bottom* and *top* the *y*-values for the minimal and maximal curve asymptotes, respectively; log IC_50_ the concentration at which 50% of the enzyme activity is inhibited (nM), *x* the logarithm of inhibitor concentration, and *hillslope* the slope factor.

Michaelis–Menten kinetics were also performed using the same GSH–CDNB conjugation assay, in the presence and absence of *x*, 10*x*, and *x*/10 of BSP (where *x* is the IC_50_ value). The *V*_max_, *K*_m_, and *k*_cat_ were determined by adding varying concentrations of GSH to 100 nM Sbh26GST. The Michaelis–Menten-enzyme kinetics (velocity as a function of the substrate) model was fitted to the data in GraphPad Prism version 8.0.2 (GraphPad Software, San Diego, CA, USA).

### 2.6. Far-UV Circular Dichroism

The secondary structural content of Sbh26GST was analyzed with far-UV circular dichroism (far-UV CD) using a Jasco J-810 spectropolarimeter with Spectra Manager software v1.5.00 (Jasco Inc., Tokyo, Japan). Samples of 2 µM protein were prepared in 5 mM Tris-HCl, pH 7.5, and 0.0002% (*w*/*v*) NaN_3_. All spectra measurements were taken at 20 °C with a bandwidth of 1 nm, a scan speed of 100 nm/min, a data pitch of 0.5 nm, and a 1 s response time for ten accumulations between 180 and 250 nm. The data were buffer corrected and normalized from millidegrees to mean residue ellipticity [*θ*] (deg.cm^2^.dmol^−1^):

$$\left[\theta \right]=\frac{100\times \theta }{\begin{array}{c} cnl \end{array}}$$

where *θ* is the CD signal in millidegrees, *c* the protein concentration (mM), *n* the number of residues, and *l* the path length (cm). Furthermore, the fractions of each secondary structural component of the protein were obtained from the DichroWeb algorithm server [25] using CONTINLL [25,26].

### 2.7. Fluorescence Spectroscopy

The 3D structure of Sbh26GST in the presence and absence of ligands was analyzed through intrinsic Trp fluorescence, whereas extrinsic ANS fluorescence was used for probing hydrophobic clefts. The spectra were measured on a Jasco FP-6300 spectrofluorometer with Spectra Manager software v1.5.00 (Jasco Inc., Tokyo, Japan) at 20 °C in triplicates. Samples were prepared in the presence and absence of 10 µM Sbh26GST and incubated for 30 min in 50 mM Tris-HCl, pH 7, and 1 mM EDTA and 1 mM DTT in the presence and absence of co-substrates 1 mM GSH as well as ligands: 100 µM of PZQ, ART, and BSP. Intrinsic Trp fluorescence was performed between 250 and 500 nm with three accumulations at 1 cm pathlength, excitation and emission bandwidth of 2.5 nm, data pitch of 1 nm, scanning speed of 200 nm/min, and excitation at 295 nm. In the case of extrinsic ANS fluorescence (200 µM), the signal was monitored between 400 and 650 nm with three accumulations at 1 cm pathlength, excitation bandwidth of 5 nm and emission of 2.5 nm, data pitch of 1 nm, scanning speed of 200 nm/min, and excitation at 395 nm. All the spectra were averaged, and buffer corrected. The fluorescence intensities in the presence of PZQ, ART and BSP were also corrected for the inner filter effect to account for any absorption of excitation (primary) and emission (secondary) light by applying the equation below [27]:

$$F_{corr}=F_{obs}\times 10^{\left(\frac{OD_{ex}+OD_{em}}{2}\right)}$$

where *F_corr_* and *F_obs_* are the corrected and measured fluorescence intensities, respectively; *OD_ex_* and *OD_em_* are the optical density values at excitation and emission wavelengths, respectively.

### 2.8. Isothermal Titration Calorimetry

The thermodynamic parameters of Sbh26GST interacting with BSP were monitored on a Nano ITC standard volume instrument (TA^®^ Instruments, New Castle, DE, USA). Sbh26GST was dialyzed against 50 mM Tris-HCl, pH 7.2, 2 mM EDTA, 0.02% (*w*/*v*) NaN_3_, and 1 mM Tris (2-carboxyethyl) phosphine (TCEP). The respective dialysates were used to prepare 1 mM BSP from a stock of 500 mM BSP in 100% (*v*/*v*) DMSO. A sample of 50 µM Sbh26GST from the dialyzed protein stock solution was then prepared with an equivalent amount of DMSO to ensure no buffer mismatch or organic solvent damage. All samples were degassed at a vacuum of 635 mmHg for ~20 min, after which the sample cell was loaded with Sbh26GST and a syringe with BSP. The experiment took place at 25 °C with a stirring rate of 310 rpm. A total of 30 injections were used with an injection volume of 5 μL every 300 s. The data were fitted using the NanoAnalyze^TM^ Software (TA^®^ Instruments, New Castle, DE, USA), whereby the integrated heats per injection were corrected for heats of dilution (HOD) and two binding models, independent and multiple sites were fitted to the data. The Gibbs free energy (∆G°) was determined using the equation below:

$$∆G{}^{\circ}=∆H{}^{\circ}-T∆S{}^{\circ}=-RT\ln K_{a}$$

where ∆*H*° is the change in enthalpy, *T* is the absolute temperature (K), and ∆*S*° is the change in entropy. Alternatively, *R* is the gas constant (8.3145 J/mol/K), *T* is the absolute temperature (K), and *K_a_* is the equilibrium affinity constant (M^−1^).

### 2.9. Thermal Shift Assay

The stability of Sbh26GST was tested through a thermal shift assay (TSA) using a CFX96 Touch-Real-Time PCR detection system (Bio-Rad Laboratories, Inc., Hercules, CA, USA) and the environment-sensitive fluorescent dye, SYPRO Orange. The quantum yield of this dye increases when bound to exposed hydrophobic patches on a thermally denatured protein. A 96-well PCR plate was filled with 25 µL samples containing 20 µM of Sbh26GST, 25 µM BSP (IC_50_), and 25 µM PZQ in dialysate with and without 0.5 mM GSH as well as 10x SYPRO Orange dye (see Figure S3 for the plate layout). Sbh26GST was dialyzed overnight at 4 °C against PBS, pH 7.2, and supplemented with 1 mM DTT and 1 mM EDTA. Thereafter, the plate was covered with a sealing film and briefly subjected to a microplate centrifuge to ensure mixing before placing it in the qPCR machine. The procedure was repeated with varying concentrations of ligands evenly distributed across the plate. The melting curve was tracked with the CFX Maestro software from 10 °C to 95 °C with 0.5 °C increments for 10 s each and plotted as relative fluorescence unit (RFU) vs. temperature (°C). Replicates were averaged and corrected with buffer blanks. Thereafter, the derivative of the plot (*-d(RFU)/dT*) was used to determine the melting temperature (*T*_m_), which was represented as the lowest point. However, final plots only show the fraction of unfolded protein that was accessible to the dye.

### 2.10. Homology Modelling and Induced Fit Ligand Docking

All computational modelling was performed on high-performance desktop servers equipped with a 16 CPU Intel core i7 5960x extreme edition (3.3 GHz, 20 M cache 16x cores), Nvidia GTX 750Ti graphics card, 32 GB DDR4-2133 MHz memory on an MSI X99 motherboard, and 264 GB RAM. Glide software (courtesy of the Centre for High Performance Computing, CSIR, Cape Town, South Africa) was utilized to perform induced fit docking (IFD) and subsequently calculate the binding free energies. Graphical visualization was performed using PyMOL software (PyMOL Molecular Graphics System, Education version).

The reference structure, the crystal structure of glutathione S-transferase from Schistosoma japonicum (PDB ID: 6RWD) [17], was recovered from the RCSB PDB and utilized to generate a Sbh26GST homology model using the SWISS-model online homology modelling tool [28]. Models were validated by PROCHECK [29]. Protein preparation was achieved using the Protein Preparation Wizard module of Schrödinger. The model was modified by correcting bond order and removing the γ-glutamyl[S-(2-iodobenzyl)cysteinyl]glycine ligand as well as water molecules within 5 Å from hydrogen atoms. The hydrogen-bonding network was optimized by sampling the orientation of water molecules using the PROPKA algorithm at pH 7.0. Water molecules forming less than three hydrogen bonds with non-water molecules were removed. The model was refined by minimization using the OPLS_2005 force field until the average RMSD reached 0.3 Å.

Structure data files of BSP (CID: 6282), PZQ (CID: 4891), and ART (CID: 68827) were extracted from the PubChem database. The 2D model of ANS was created using Biovia Draw. Ligand preparation was achieved using the LigPrep and Epik modules of Schrödinger. These tools were used to desalt the ligand, generate possible tautomeric states at pH 7.0 ± 2 (experimentally determined optimal pH), accurately predict the pK_a_ of these states, correct chirality and perform energy minimization using the OPLS_2005 force field.

Induced fit ligand docking was used to predict the binding of ANS, BSP, PZQ, and ART to the Sbh26GST model. The entire protein dimer (chains A and B) was mapped as the binding site since the enzyme exhibits three possible binding sites [15]: the GSH binding site (G-site), the hydrophobic substrate-binding site (H-site), and a binding site located at the dimer interface (L-site). In the induced fit ligand docking protocol an implicit solvent model using the OPLS_2005 force field was applied, in addition to ring conformational sampling with a 2.5 kcal/mol energy barrier and a non-planar conformation penalty on amide bonds. The scaling for both receptor and ligand was set at 0.5 with a maximum of 20 allowable poses per ligand. The Prime Refinement module was then used to further refine models in which the residues were within 5.0 Å of the docked ligand. Thereafter, the Prime Energy algorithm was utilized to rank the refined protein-ligand complexes. The receptor structures within 30.0 kcal/mol of the minimum energy structure were submitted for a final round of Glide docking and scoring. Each ligand was re-docked into every single refined low-energy receptor structure in the subsequent second docking step using the default Glide XP settings.

## 3. Results

### 3.1. Expression and Purification of Sbh26GST

Given the well-studied nature of GSTs, the induction trial was not extensive. The optimal conditions used for the overexpression of Sbh26GST were induction with 0.5 mM IPTG for approximately six hours at 30 °C. Most of the recombinant protein was found to be in the soluble fraction of the cell lysate. The molecular weight of the monomer is ~25 kDa, which corresponds to the theoretical subunit weight of each polypeptide. Cell cultures that were not induced with IPTG did not show any significant amount of protein with a similar size (results not shown).

**Figure 2:**
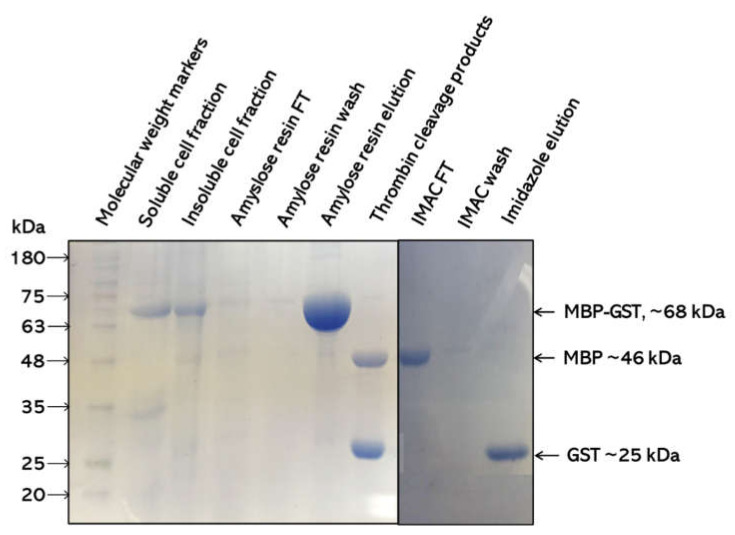
SDS–PAGE analysis of Sbh26GST over-expression and purification. Cell lysate from the IPTG induction was separated into the insoluble pellet and soluble supernatant fractions. The supernatant was further subjected to an amylose resin column that binds maltose-binding protein (MBP). The flow-through (FT) had unbound protein, and weakly bound proteins were removed via several washes before elution with maltose. The recovered protein was cleaved with thrombin and purified with IMAC.

The purification procedure of using two affinity chromatography methods was effective in that pure protein with >95% homogeneity was obtained (Figure 2). The MBP-amylose purification showed that the MBP from the vector interacted with amylose, thus isolating the pseudo-recombinant protein with ˃95% purity. The recovered protein was completely cleaved with thrombin into the MBP protein and His-tagged Sbh26GST. The second IMAC purification was needed to separate the thrombin cleavage products whereby only the His-tagged Sbh26GST was bound to the Ni^2+^ column. The protein yield was ~2 mg/g of wet cells per liter of culture.

### 3.2. Characterization of Sbh26GST Using GSH–CDNB Conjugation Assay

**Figure 3:**
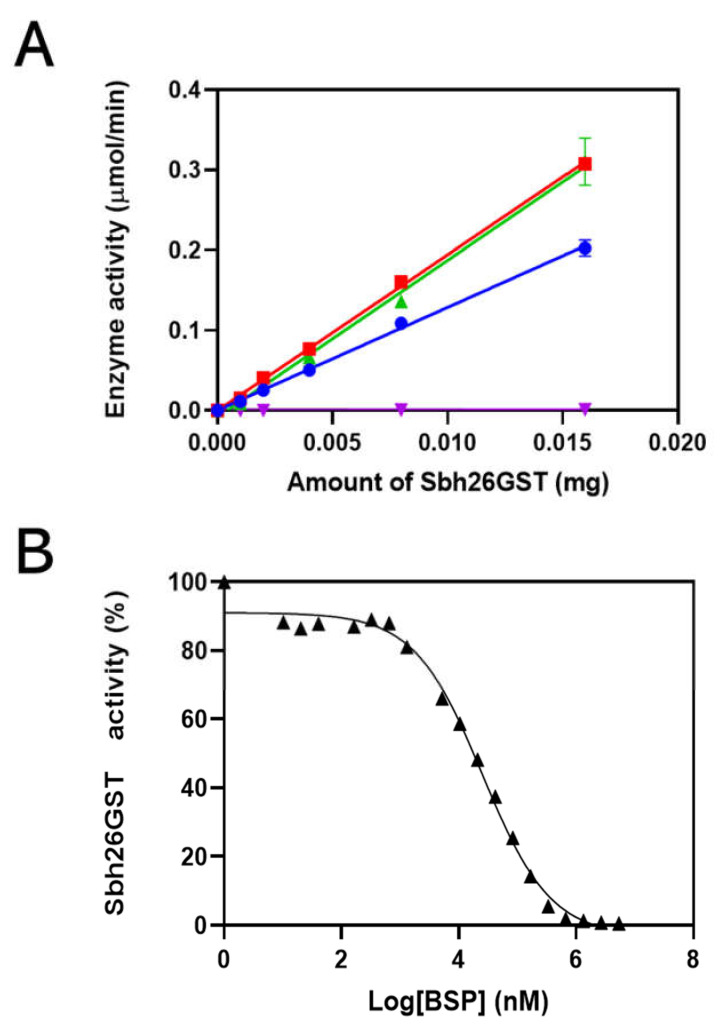
Specific activity analysis of Sbh26GST. (**A**) The specific activity was determined by varying the concentration of Sbh26GST when performing the GSH–CDNB assay and was found to be 13 µmol/min/mg in the absence of any ligand (**blue**). In the presence of 0.5 mM BSP (**purple**) the activity decreased to 0.07 µmol/min/mg, whereas in the presence of 0.5 mM PZQ (**red**) or 0.5 mM ART (**green**), the activity increased insignificantly to 19 µmol/min/mg. (**B**) the concentration of BSP was varied while performing the CDNB assay with 100 nM Sbh26GST to determine its IC_50_, which was found to be 27 µM.

The specific activity of Sbh26GST showed that this enzyme converts reactants to products at 13 µmol/min/mg (Figure 3A). In the presence of 0.5 mM BSP, activity decreases to 0.07 µmol/min/mg. However, the activity increases insignificantly in the presence of PZQ and ART to 19 µmol/min/mg. The ligand that showed inhibition, BSP, was further used for IC_50_ analysis, as shown to be 27 µM when there is 100 nM of Sbh26GST in Figure 3B. Furthermore, the kinetics of the reaction was studied to gain knowledge about the performance of Sbh26GST. The *k*_cat_, *K*_m_, and *V*_max_ were altered by 27 µM of BSP from 9.8 s^−1^,120 µM, and 0.19 µmol/min to 1.2 s^−1^, 36.5 µM, and 0.027 µmol/min, respectively (Table 1). Essentially, BSP decreased the reaction rates, whereas PZQ and ART increased the reaction rate.

### 3.3. Structural Analysis of Sbh26GST

**Figure 4:**
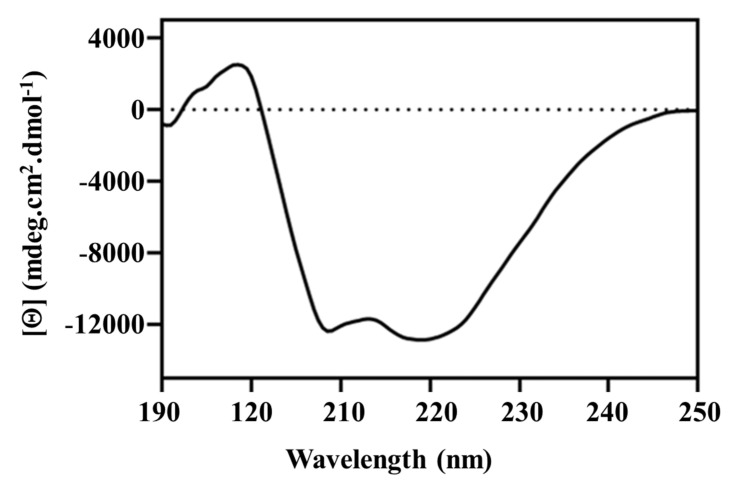
Secondary structural content of Sbh26GST determined via far-UV CD. The spectrum was obtained using 2 µM protein in 5 mM Tris-HCl, pH 7.5, and 0.0002% (*w*/*v*) NaN_3_.

The secondary structural content of pseudo-recombinant Sbh26GST was further deconvoluted via the DichroWeb-CONTILL algorithm (data not shown). The CD spectrum exhibits negative minima at both 219 and 208 nm, which is characteristic of α-helical dominant proteins such as GSTs and agrees with the DichroWeb calculations (Figure 4).

**Figure 5:**
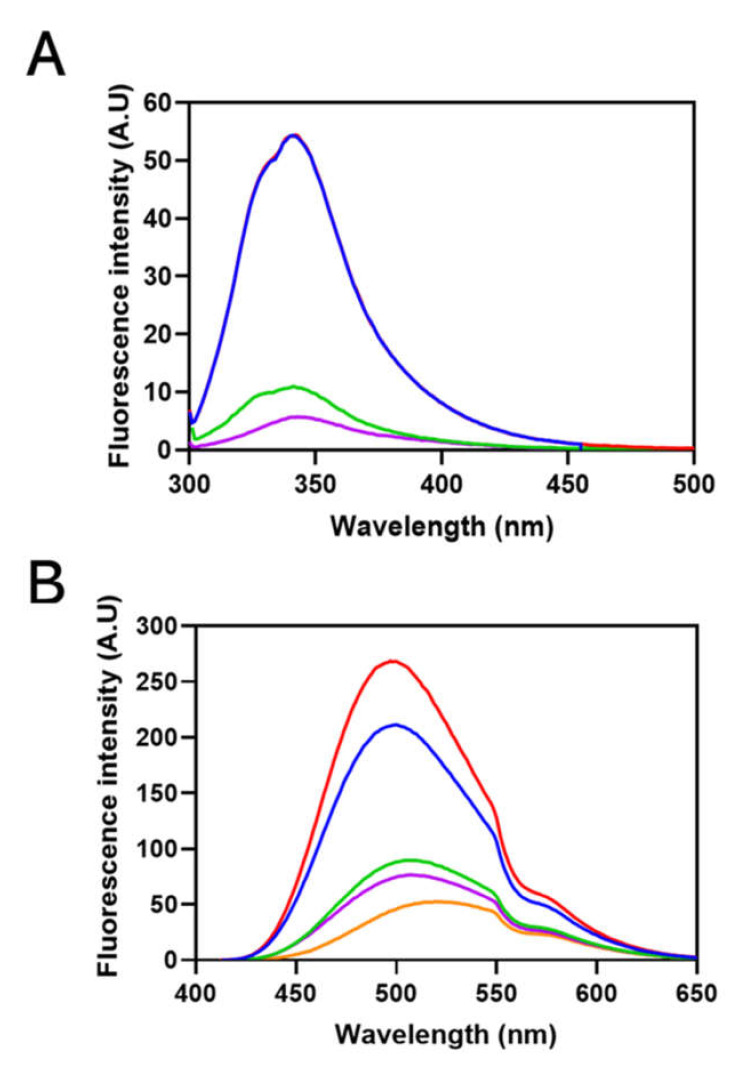
Fluorescence spectroscopy of the ligandin activity of Sbh26GST towards ANS, ART, PZQ, and BSP. (**A**) Intrinsic tryptophan fluorescence emission spectra of 10 µM Sbh26GST in 50 mM Tris-HCl, pH 7, 1 mM EDTA, and 1 mM DTT with bound 1 mM GSH (**blue**), was excited at 295 nm. The effects of ligands on the spectrum were evaluated by adding 100 µM of BSP (**purple**), ART (**green**), and PZQ (**red**) separately. (**B**) Extrinsic ANS binding fluorescence emission spectra of Sbh26GST. A 10 µM Sbh26GST in 50 mM Tris-HCl, pH 7, 1 mM EDTA, and 1 mM DTT with bound 1 mM GSH was excited at 395 nm in the presence of 200 µM ANS as well as various ligands. The **orange** line represents free ANS which was compared to ANS bound Sbh26GST (**blue**) in the presence of BSP (**purple**), ART (**green**), and PZQ (**red**) separately.

Intrinsic Trp fluorescence of Sbh26GST was performed in the presence and absence of 100 µM ligands. Sbh26GST contains four Trp residues within each subunit, all situated in the *N*-terminus thioredoxin-like domain with Trp206 closest to the active site. The presence of PZQ did not affect the local environment of the Trp residues in Sbh26GST, displaying an emission peak at 341 nm. However, ART and BSP decreased the quantum yield of Trp residues by 80 and 90%, respectively, in addition to an insignificant redshift of 2 nm (Figure 5A). A similar trend was observed in the presence of the natural substrate GSH (1 mM) and GTX, since ligands of interest were still able to bind the protein.

ANS was used as a probe for hydrophobic patches on the pseudo-recombinant Sbh26GST, since a hypsochromic shift would indicate the occurrence of binding. In the binding assay (Figure 5B), the quantum yield of sole 200 µM ANS significantly increased in the presence of Sbh26GST. Furthermore, the wavelength of the emission maximum underwent a blueshift from 518 nm to 493 nm. PZQ did not have any significant effect on the spectrum. However, the presence of BSP and ART decreased the quantum yield of the ANS: Sbh26GST complex in addition to a maximum emission redshift to 502 nm due to ANS displacement.

### 3.4. Thermodynamic Parameters of the Interactions between Sbh26GST and BSP

**Figure 6:**
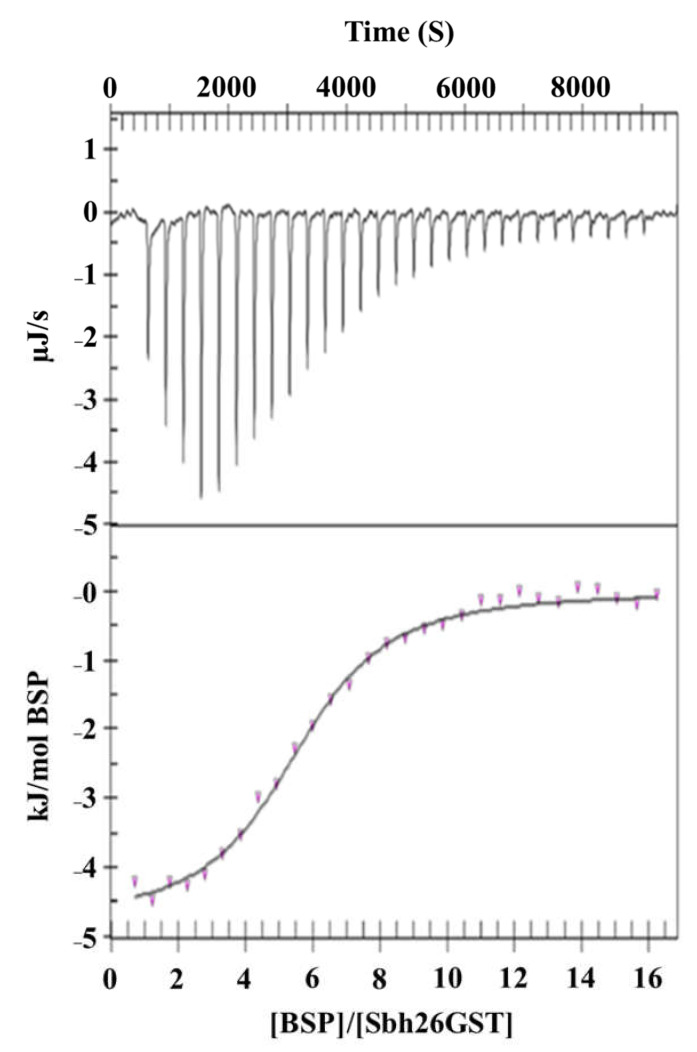
Isothermal titration calorimetry of the interaction between BSP and Sbh26GST. The study was performed at 25 °C whereby 1 mM of BSP was titrated into 50 µM Sbh26GST. All samples were buffered by 50 mM Tris-HCl, pH 7.2, 2 mM EDTA, 0.02% (*w*/*v*) NaN_3_, and 1 mM Tris (2-carboxyethyl) phosphine (TCEP). The data were fitted using the independent binding model algorithm implemented in the NanoAnalyze software.

The thermodynamics of the interaction between BSP and Sbh26GST in the presence and absence of GST were studied via ITC to gain insights into the energy profile of the binding sites. BSP was titrated into Sbh26GST, all in a buffer with or without bound GSH to the protein at 25 °C until saturation was reached, as shown in Figure 6. The binding isotherm demonstrated in Table 2 indicates that the reaction is exothermic (0 > ΔH°). Given the possibility of BSP binding to the single L-site and/or the two independent H-site per GST dimer, the independent model and multiple site models were individually fitted to the data. The independent model showed that one molecule of BSP spontaneously (0 > Δ*G°*) binds at the dimer interface tightly with a *K*_d_ of 35 nM driven by entropy (0 < Δ*S*°). This trend is also observed in the multiple site model as indicated by thermodynamic parameters associated with *K*_d_ of 1 nM (site 1). However, the latter model has an additional ability of two BSP molecules binding at each H-site, resulting in a total stoichiometry of five. Moreover, BSP binding at the H-site with *K*_d_ of 54 nM is not enthalpically favorable but entropically driven.

### 3.5. Thermal Stability of Sbh26GST

**Figure 7:**
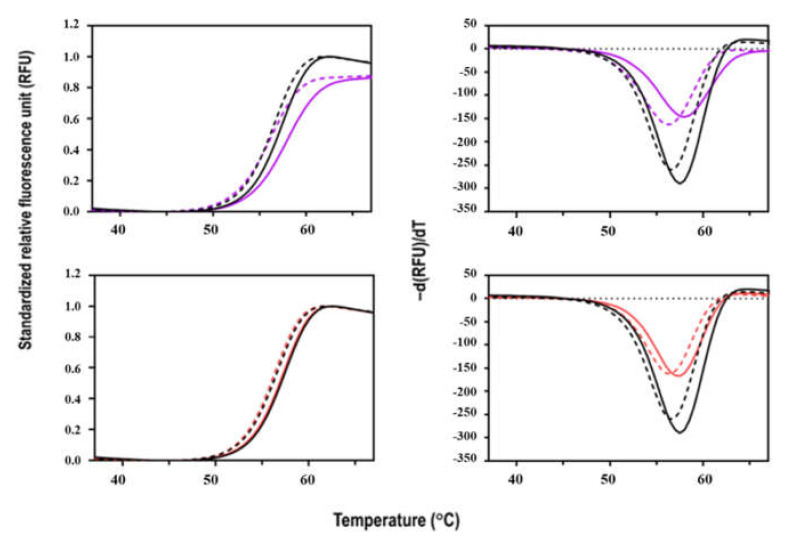
Thermal stability profile of Sbh26GST in the presence and absence of BSP and PZQ. Fluorescence thermal shift assay was carried out in phosphate-buffered saline with and without 1 mM GSH in the presence of Sbh26GST (10 µg) and Sbh26GST:ligand (10 µg:5 µM) combinations. This was screened in 16 replicates, and the data points averaged to generate individual thermal melt curves for data comparison (with the blank subtracted). The left panel shows the standardized RFU vs. temperature (°C), while the right panel shows the derived -*d(RFU)/T* vs. temperature (°C). The position of the minimum indicates the *T*_m_ (°C) of each profile. The black dashed profile represents **Sbh26GST** alone; the bold black profile **Sbh26GST:GSH**, the dashed purple profile **Sbh26GST:BSP**, the bold purple profile **Sbh26GST:GSH:BSP**, the dashed red profile **Sbh26GST:PZQ**, and the bold red profile **Sbh26GST:GSH:PZQ**.

The denaturation of Sbh26GST with increasing temperature was coupled with SYPRO Orange dye binding in a fluorescence thermal shift assay. The dye has a binding propensity for hydrophobic patches that result in an increased quantum yield. The thermal denaturation profile of Sbh26GST follows a cooperative unfolding curve (Figure 7). The protein is stable at room temperature, but with increasing temperature, the protein unfolds as shown by the single transition that peaks at ~60 °C. The more hydrophobic regions are exposed, the more the dye occupies the regions. When saturation is reached, the dye gets quenched due to aggregation, as shown by decreasing fluorescence, making the plot asymmetrical. Furthermore, Sbh26GST is more stable in the presence of GSH, as demonstrated by the slight *T*_m_ increase from apo 56.5 ± 0.35 °C to 57.5 ± 0.46 °C in 0.5 mM GSH. The apo *T*_m_ is comparable with other GSTs of similar structure such as Sj26GST (53.1 °C) [30].

### 3.6. Theoretical Modelling of Sbh26GST Interacting with ANS, ART, PZQ, and BSP

**Figure 8:**
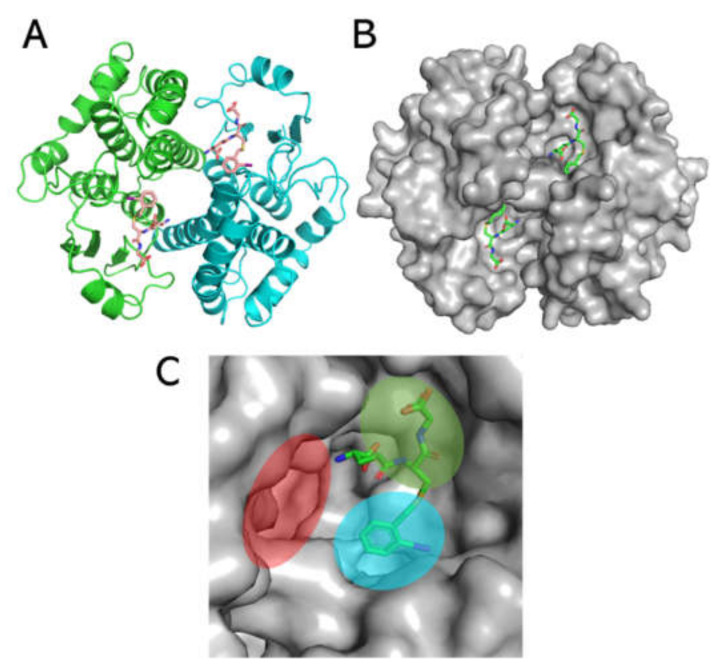
Homology model of pseudo Sbh26GST showing the canonical GST fold. (**A**) Ribbon representation of the model showing the presence of γ-glutamyl[S-(2-iodobenzyl)cysteinyl]glycine (stick formation). The two subunits are colored **green** and **cyan**. (**B**) Surface representation of the model showing the active site pockets of this multi-substrate enzyme. (**C**) One of the active sites in a monomeric unit. The 2-iodobenzyl moiety is oriented towards the hydrophobic binding site (H-site) shaded **cyan**; the gamma-glutamyl-cysteinyl-glycine moiety is at the GSH binding site (G-site), shaded **green**. The dimer interface, also referred to as the ligand site (L-site), is shaded **red**.

To simulate the ligandin activity of Sbh26GST towards the ligands of interest, a homology model was designed using the Swiss Model and induced-fit ligand docking performed via Schrodinger Maestro v12.0. The homology model was generated on the template of the *S. japonicum* 26-kDa GST (PDB: 1M9B), which affirms that the sequence strongly correlates (Figure S4) with Sbh26GST. The model was generated with γ-glutamyl[S-(2-iodobenzyl)cysteinyl]glycine bound to the active site (Figure 8), with the 2-iodobenzyl moiety oriented towards the H-site and the remaining part of the ligand oriented towards the G-site. The root-mean-square-deviation (rmsd) between the template and the model is < 0.002 Å, and all side chains show good stereochemical properties, since none reside in the disallowed region of the Ramachandran plot (Table 3 and Figure S5) and only 0.5% occupy the generously favorable region of the plot.

**Figure 9:**
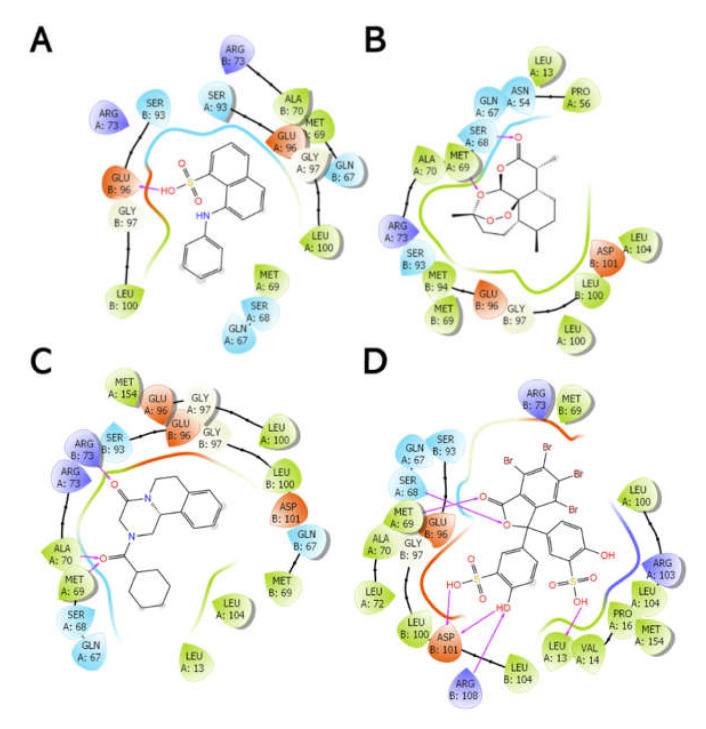
Two-dimensional interaction diagrams between the Sbh26GST dimer interface amino acids and hydrophobic molecules, namely, ANS (**A**), ART (**B**), PZQ (**C**), and BSP (**D**). The **purple** lines represent H-bond interactions. This image was generated using the ligand interaction diagram implemented in Maestro v12.0.

**Figure 10:**
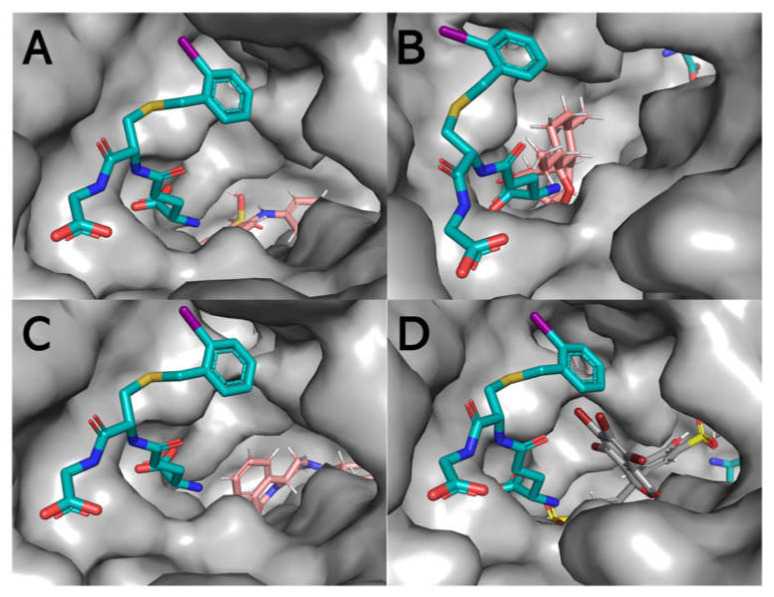
Surface representation of the active site of one of the subunits displaying the binding of hydrophobic molecules, in particular, ANS (**A**), ART (**B**), PZQ (**C**), and BSP (**D**) concerning the position of the γ-glutamyl[S-(2-iodobenzyl)cysteinyl]glycine (cyan stick formation) ligand, which makes contact with both the G-site and the H-site. ANS, ART, PZQ, and BSP appear to reside primarily within the L-site of the structure. The image was generated using PyMOL (PDB: 1M9B).

The entire protein was mapped for induced fit ligand docking (IFD) instead of selecting a defined region, such that the docking is performed blind. The IFD results showed that all four ligands preferably bind at the dimer interface (L-site) with varying degrees of Emodel energy (kcal/mol) and in several poses (Figure S6). BSP appears to form the most stable and strongest interactions with the protein, since the Emodel energy was the lowest (−120.18 ± 6.67 kcal/mol) in comparison with ANS (−49.54 ± 3.79 kcal/mol), ART (−65.74 ± 3.71 kcal/mol), and PZQ (−72.10 ± 5.02 kcal/mol). In addition to the Emodel energy, BSP only exhibits three possible binding conformations relative to ANS, ART, and PZQ, each displaying 19, 18, and 15 poses, respectively (Figure S6). Using the top-scoring pose (based on the Emodel energy) as the benchmark, Figure 9 shows that BSP binds with the most significant H-bonds within the L-site near the H-site (~2.3 Å). The distance is close enough to prevent or alter the conformational space for a hydrophobic (non-substrate)-binding pocket (Figure 10 and Supplementary Figure S7).

## 4. Discussion

GSTs are known for interacting with diverse electrophilic substrates in phase II detoxification processes [31]. In schistosomes, there are two isoforms to compensate for the lack of alternatives, such as cytochrome P-450-mediated xenobiotic metabolism [15]. However, studies on *Schistosoma haematobium* GSTs, one of the species which infect humans, are limited due to the incomplete sequence of Sh26GST. Furthermore, this species is zoonotic and displays 100% sequence similarity to *S. bovis*, a species known to infect domesticated animals/livestock. This establishes the possibility that *bovis* and *haematobium* are predisposed to forming genetic hybrids that are usually heterotic [7]. Therefore, the partial 26-kDa GST sequence from *bovsis* was rationally used as a template to complete the full-length 26-kDa GST, despite the 12 missing *N*-terminus amino acids. The complete pseudo-Sbh26GST sequence was then theoretically derived by using well-known closely related species to fill in the missing first 12 amino acids’ residues in the ORF of Sh26GST/Sb26GST.

Pseudo-Sbh26GST was successfully cloned and over-expressed in an *E. coli* system at the *N*-terminus, with the MBP assisting in overall solubility and downstream purification of the recombinant protein. Furthermore, a 6×His-tag was added to the *N*-terminus for ease of purification (it yielded homogenously pure Sbh26GST). Thrombin cleavage site was incorporated upstream of the His-tag to facilitate the cleavage of the MBP-tag from the recombinant protein. In several studies involving MBP-fusion proteins, factor-Xa was not highly efficient in separating the MBP-tag from the recombinant protein, since factor-Xa tends to incompletely cleave the tag compared to thrombin. The His-tag was left intact, as this has a negligible effect on the function of GSTs based on previous studies. The catalytic activity of Sbh26GST was assessed with the standard GSH–CDNB conjugation assay [32,33]. GSTs catalytically function as obligate homodimers with one active site per monomer that comprises the G-site for binding GSH and the H-site for hydrophobic substrates. Moreover, the dimer interfaces create an additional electrophilic non-substrate L-site. This conformation contributes to the tertiary structure and offers some stability [34,35]. Pseudo-Sbh26GST was active with a rate of 13 µmol/min/mg, which is comparable to previous studies involving 26-kDa GST from *Schistosoma japonicum* [16].

Furthermore, the activity of this construct was slightly enhanced by PZQ and ART. However, BSP completely abolished the activity of the pseudo enzyme. The observed inhibition of the enzyme by BSP was further probed to derive the IC_50_ value of BSP towards the enzyme. The value was within the µM range, comparable with our previous study’s observations [16]. The ligandin activity of the pseudo enzyme was further studied to obtain the kinetic parameters of the enzyme to gain insights into the potential binding sites of the studied ligands and how this activity may affect enzyme efficiency. Sbh26GST displayed a slightly higher affinity for GSH in the presence of BSP but a decrease in catalytic activity towards CDNB, thus making the enzyme inefficient. On the other hand, ART and arguably PZQ increased activity. All ligands were non-competitive with GSH, which binds to the conserved G-site but adjacent to the H-site, which accommodates diverse electrophiles. The interaction with the ligands (possibly within the L-site) distorted the conformation of the H-site, where CDNB is known to bind, hence the observed changes. The kinetics of CDNB interaction with the pseudo enzyme were not investigated further as this is not a natural substrate of the enzyme. Our previous studies showed that a Michaelis–Menten curve does not attain saturation within practical concentration ranges of CDNB (5 mM).

The observations made on the catalytic and ligandin activities led us to further probe the structure of Sbh26GST to understand the impacts of the ligands on the structural attributes of the enzyme. The secondary structural content of Sbh26GST was established through far-UV CD, which agrees with the recognized secondary structure of cytosolic GSTs, which is predominantly α-helical [36,37]. Furthermore, the tertiary structure of Sbh26GST was assessed using intrinsic Trp fluorescence spectroscopy. The enzyme contains four Trp residues per monomer, all of which are close to each other (2.3–5 Å) and the G-site. Thus, interactions within the active site or any interaction that modulates the active site topography is assumed to affect the local Trp environment, which will, in turn, alter the spectral property of these residues. As expected, the Trp fluorescence intensity of the enzyme was remarkably decreased in the presence of ART and BSP. This indicates that these two ligands may have altered the local Trp environment.

Furthermore, ANS has previously been shown to bind the H-site of GSTs with moderate affinity [38,39]. The binding of ANS to apo Sbh26GST showed a higher fluorescence quantum yield at 500 nm than free ANS at 520 nm. Moreover, this is accompanied by a blue wavelength shift as observed in other GSTs [39,40]. This result does not preclude the possibility of ANS accessing more than one binding pocket in the protein. Alteration of the spectral properties of ANS in the presence of the ligands may indicate that the ligands could modulate the enzyme’s active site, possibly the H-site. The results confirmed that BSP and ART out-competes ANS for the binding pockets, which may corroborate the effect of BSP and ART on the intrinsic Trp residues. However, we could not logically explain why ANS fluorescence yield increased in the presence of PZQ, despite the absence of the inner-filter effect by the ligands in the presence of ANS. On the other hand, ART and BSP displaced ANS by ~ 3 folds, suggesting that these compete for the same site. One also needs to note that the H-site is highly promiscuous for electrophiles, thus allowing weak and transient interactions to occur [34]. ART and BSP bind in a manner that still allows some ANS to bind. Therefore, these ligands are more inclined to interact with the L-site, which is not entirely hydrophobic.

Having established that BSP strongly interacts with the enzyme, resulting in non-competitive inhibition of GSH–CDNB conjugation activity of the enzyme, it was thought crucial to characterize the thermodynamics of this ligandin activity. These interactions are usually stabilized by non-covalent forces, such as van der Waals contacts, hydrogen bonding, and ionic and hydrophobic interactions. The interaction between BSP and Sbh26GST proved to be spontaneous, driven both enthalpically and entropically with preferential binding to the dimer interface over the H-site. Considering the hydrophobic nature of BSP, the entropic contribution can be attributed to the hydrophobic interaction between this ligand and the enzyme. Despite BSP making contacts with the protein, which reduces its conformation freedom, de-solvation of the binding site increases entropy, thus rendering the interaction favorable.

Furthermore, the indirect binding affinity (*k*_d_) between Sbh26GST and BSP is again observed to be in the moderate micromolar range with a stoichiometry of one molecule per dimer. This supports our postulation of BSP having the possibility of binding at the L-site of Sbh26GST, thus rationalizing using this drug as a GST inhibitor. The thermodynamics of PZQ interaction with Sbh26GST could not be established due to the high heat of dilution generated by ethanol, which is required to solubilize PZQ. On the same note, ART did not solubilize in either DMSO or ethanol at concentrations useable for ITC.

Protein–ligand interactions impact the conformational stability of the protein [41]. We expected BSP to affect the stability of the pseudo enzyme based on the postulation of possible, stable interaction it makes, extrapolated from the inhibition, IC_50_, and thermodynamic studies. Thus, a fluorescence-based thermal shift assay was used as an all-purpose method for identifying inhibitors of Sbh26GST from compound libraries to probe the conformational stability of the enzyme in the presence of BSP. The results showed that BSP slightly stabilized the protein, especially in the presence of GSH. These results further support that BSP could potentially be modified as a drug candidate, since this alteration may affect conformational dynamics, hence enzyme functionality. There is no currently published data we could have used to compare our studies. The implication is that any strong and stable interaction within the L-site that results in stabilization of this enzyme may also restrict or alter the conformational dynamics of the enzyme, resulting in inhibition.

Theoretically, however, we must use computational modelling to support and complement our results obtained empirically. The primary focus of this study is the ligandin activity of this pseudo enzyme; hence, we used molecular docking based on the induced fit ligand docking algorithm implemented in Schrödinger Maestro v12.0. This algorithm simulates the interaction between a macromolecule and a small molecule ligand, because the conformation of the receptor modifies to accommodate the rigid ligand. The stability of this interaction depends mainly on the scoring function and the number of poses of the ligand within the receptor. However, we needed to generate a theoretical model of the pseudo protein before applying molecular docking. Using the Swiss Model algorithm, we developed a homology model of the pseudo enzyme based on the Sj26GST template, the top-scoring template. The model is likely accurate, since the sequence identity between the template and the model is >80%, and there was no residue in the least favorable regions of the Ramachandran plot. The root-mean-square-deviation between the template and the model was <0.02 Å, indicating a strong correlation with the template structure.

The IFD showed that the interaction between Sbh26GST and the four ligands are energetically favorable and that all ligands preferably bind at the dimer interface. This has been demonstrated in previous studies that the L-site is an optional binding site in Sj26GST [15,16]. However, BSP appears to be a stronger binder than PZQ, ART, and ANS based on the number of interactions with the side chains in the L-site, which explains the lowest Emodel energy compared to PZQ, ART, and ANS. Additionally, BSP had the lowest number of poses (three), indicating that this ligand can adopt a limited number of conformations within the dimer interface. The results affirm the spectroscopy results which showed that, although ANS interacts with Sbh26GST, the dye was displaced by ART and BSP. However, the docking results indicated that PZQ binds slightly tighter than ANS, but could not explain the inability of PZQ to displace ANS. IFD demonstrates that interaction of BSP within the L-site may interfere with the binding of CDNB in the H-site active site pocket. Nonetheless, IFD gives us an insight into the inhibitory properties of BSP and the lack thereof for ART and PZQ. This study supports BSP as a prospective drug candidate that can be modified as an effective inhibitor against Schistosoma GST.

## 5. Conclusions

In the absence of a complete ORF encoding the putative 26-kDa isoform of GST in *bovis* and *haematobium* species of *Schistosoma*, we have engineered a pseudo version of the 26-kDa GST derived from sequence analysis of the fragments of this enzyme in these two species. This enzyme encodes a Tyr residue at position 7, which is the catalytic residue found in most cytosolic eukaryotic GSTs. The enzyme displayed characteristics of the canonical GST and can be inhibited by BSP. One major limitation of this study is the lack of empirically structural data to support some of our postulates. However, we are currently optimizing the recombinant expression of this pseudo enzyme to obtain higher quantities of the protein for atomic resolution crystal structure studies. One major highlight of this study is that BSP, a diuretic dye commonly used in humans to assay liver or kidney function, has the potential to be repurposed as a possible anti-helminthics. This is based on the foundation that BSP does not inhibit human GSTpi, GSTα, and GSTmu (data to be published elsewhere) to the same degree as Schistosoma GST. We furthered our studies using computational modelling to corroborate our empirical results.

## Figures and Tables

**Table 1:** Kinetic parameters of GSH towards Sbh26GST.

Kinetic Parameter	Value ± SD		
	No Ligand	ART	BSP
V_max_ (μmol/min)	0.19 ± 0.020	0.32 ± 0.080	0.03 ± 0.001
*K*_m_ (μM)	120.00 ± 0.030	250.00 ± 0.090	36.50 ± 0.006
*k*_cat_ (S^−1^)	9.80 ± 0.056	39.00 ± 0.011	1.20 ± 0.130
*k*_cat_/*K*_m_ (M/s)	0.08 ± 0.041	150.00 ± 0.012	0.03 ± 0.003

**Table 2:** Thermodynamic parameters from ITC analysis of the interaction between BSP and Sbh26GST. The study was performed at 25 °C whereby 1 mM of BSP was titrated into 50 µM Sbh26GST. All samples were buffered by 50 mM Tris-HCl, pH 7.2, 2 mM EDTA, 0.02% (*w*/*v*) NaN_3_, and 1 mM Tris (2-carboxyethyl) phosphine (TCEP). Two binding models were used to fit the binding isotherm.

Thermodynamic Parameter	Independent ^1^ Binding Model (Value ± SD)	Multiple Site Binding Model (Value ± SD)
Stoichiometry (n_1_)	1.13 ± 0.02	1.12 ± 0.05
Stoichiometry (n_2_)		4.83 ± 0.70
∆*H*°_1_ (kJ/mol)	−23.35 ± 0.28	−24.20 ± 0.33
∆*H*°_2_ (kJ/mol)		0.53 ± 1.20
∆*S*°_1_ (J/mol·K)	26.12 ± 0.02	91.10 ± 0.06
∆*S*°_2_ (J/mol·K)		140.90 ± 0.90
∆*G*°_1_ (kJ/mol)	−31.14 ± 0.02	−51.37 ± 0.07
∆*G*°_2_ (kJ/mol)		−41.48 ± 0.87
*K_d_*_1_ (nM)	35.07 ± 0.01	1.00 ± 0.01
*K_d_*_2_ (nM)		53.83 ± 0.81

^1^ The preferred binding model.

**Table 3:** The number and percentage of residues within the four regions of the Ramachandran plot (Figure S5).

Ramachandran Plot Region	Number of Residues	Percentage (%)
Most favored	352	94.60
Additional allowed	18	4.80
Generously allowed	2	0.50
Disallowed	0	0.00

## Supplementary Materials

The following are available online at https://www.mdpi.com/article/10.3390/biom11121844/s1, Figure S1: The Clustal Omega multiple sequence alignment data showing 99.9% sequence conservation between the gene fragment encoding putative 26-kDa GST from S. haematobium (EBI ID KAF1314627) and S. bovis (EBI ID RTG84353); Figure S2: 2D illustration of the structures of the four ligands used in the study; Figure S3: The 96-well plate plan used for the fluorescence-based thermal shift assay on the effect of PZQ and BSP on the stability of the pseudo Sbh26GST; Figure S4. Computation of a 3-dimensional structural alignment between pseudo Sbh26GST and Sj26GST using the Expresso Alignment algorithm; Figure S5. Ramachandran plot analysis of Sbh26GST; Figure S6. Ribbon representation of the 3D homology model of Sbh26GST indicating the preferred binding site for ANS, ART, PZQ and BSP based on the induced fit ligand docking implemented in Schrödinger Maestro v12.0; Figure S7. Stick representation of the proximity between the ligands bond within the dimer interface and the active sites occupied by the glutathione moiety in the G-site and the 2-iodobenzyl moiety occupying the H-site.

## Abbreviations

The following abbreviations are used in this manuscript:

PZQ	Praziquantel
ART	Artemisinin
ANS	8-anilino-1-naphthalenesulfonate sulfonate
BSP	Bromosulfophthalein
GST	Glutathione transferase
IFD	Induced fit docking
CD	Circular dichroism
GSH	Glutathione
CDNB	1-Chloro-2,4-dinitrobenzene
MBP	Maltose binding protein
IPTG	Isopropyl β-D-1-thiogalactopyranoside
ORF	Open reading frame
DTT	Dithiothreitol
EDTA	Ethylenediaminetetraacetic acid
