# Supplementary material for: Engineering a Pseudo-26-kDa Schistosoma Glutathione Transferase from bovis/haematobium for Structure, Kinetics, and Ligandin Studies
Source: Biomolecules. 2021 Dec 7;11(12):1844. doi: 10.3390/biom11121844 (PMC8699318; doi:10.3390/biom11121844)
Supplement: Supplementary file 1 [file biomolecules-11-01844-s001.zip › biomolecules-1428722-supplementary.pdf]

Engineering a pseudo-26-kDa *Schistosoma* glutathione transferase from *bovis/haematobium* for structure, kinetics and ligandin studies

Neo Padi, Blessing Oluebube Oyiogu, Chinyere Aloke, Vanessa Meyer and Ikechukwu Achilonu*

Supplementary Section

```
CLUSTAL O(1.2.4) multiple sequence alignment


ENA|RTG84353|RTG84353.1         CTTGTACAACCGACTCGTCTTCTTTTGGAATATGTTGGCGAAGTATATGAAGAGCGTTTG   60
ENA|KAF1314627|KAF1314627.1     CTTGTACAACCGACTCGTCTTCTTTTGGAATATGTTGGCGAAGTATATGAAGAGCGTTTG   60
                                ************************************************************

ENA|RTG84353|RTG84353.1         TATGATCGCAATGATGGTGATGTCTGGCGAAACGAAAAGTTCAATTTAGGTCTGGAATTT   120
ENA|KAF1314627|KAF1314627.1     TATGATCGCAATGATGGTGATGTCTGGCGAAACGAAAAGTTCAATTTAGGTCTGGAATTT   120
                                ************************************************************

ENA|RTG84353|RTG84353.1         CCTAATCTACCTTATTATATTGATGGTGATGTTAAATTAACACAGTCTATGGCTATTTTA   180
ENA|KAF1314627|KAF1314627.1     CCTAATCTACCTTATTATATTGATGGTGATGTTAAATTAACACAGTCTATGGCTATTTTA   180
                                ************************************************************

ENA|RTG84353|RTG84353.1         CGTTATATAGCTGATAAACACAATATGTTGGGTGGTTGTCCCAAAGAACGTGCAGAAATT   240
ENA|KAF1314627|KAF1314627.1     CGTTATATAGCTGATAAACACAATATGTTGGGTGGTTGTCCCAAAGAACGTGCAGAAATT   240
                                ************************************************************

ENA|RTG84353|RTG84353.1         TCTATGCTTGAGGGAGCCATTTTGGATATCAGACTTGGTGTTTCAAGAATTGCATATAAT   300
ENA|KAF1314627|KAF1314627.1     TCTATGCTTGAGGGAGCCGTTTTGGATATCAGACTTGGTGTTTCAAGAATTGCATATAAT   300
                                ******************.*****************************************

ENA|RTG84353|RTG84353.1         AAAGAATTTGAAACTCTCAAAGTTGGTTTTCTCAATCAACTCCCTGGAATGCTGAAAATG   360
ENA|KAF1314627|KAF1314627.1     AAAGAATTTGAAACTCTCAAAGTTGGTTTTCTCAACCAACTCCCTGGAATGCTGAAAATG   360
                                *********************************** ************************

ENA|RTG84353|RTG84353.1         TTTGAAAATCGTTTATCTCACAAAATATATTTGAATGGTGATAATGTGACTCATGTTGAC   420
ENA|KAF1314627|KAF1314627.1     TTTGAAAATCGTTTATCTCAC---------------------------------------   381
                                *********************

ENA|RTG84353|RTG84353.1         TTCATGCTGTACGACGCTCTTGATGTAGTTTTATACATGGACCCAAAGTGCTTGGATGCA   480
ENA|KAF1314627|KAF1314627.1     ------------------------------------------------------------   381


ENA|RTG84353|RTG84353.1         TTTCCAAAACTAATTAGTTTCAAACAGCGTATAGAAAATTTACCACCAATCAAGAACTAC   540
ENA|KAF1314627|KAF1314627.1     ------------------------------------------------------------   381


ENA|RTG84353|RTG84353.1         CTGAATTCTGACAGGCACATAAAATGGCCTCTGCAAGGCTGGTCCGCCATTTTTGGTGGT   600
ENA|KAF1314627|KAF1314627.1     ------------------------------------------------------------   381


ENA|RTG84353|RTG84353.1         GGAGATGCTCCTCCAAAATAA   621
ENA|KAF1314627|KAF1314627.1     ---------------------   381
```

Figure S1: The Clustal Omega multiple sequence alignment data showing 99.9% sequence conservation between the gene fragment encoding putative 26-kDa GST from *S. haematobium* (EBI ID KAF1314627) and *S. bovis* (EBI ID RTG84353).

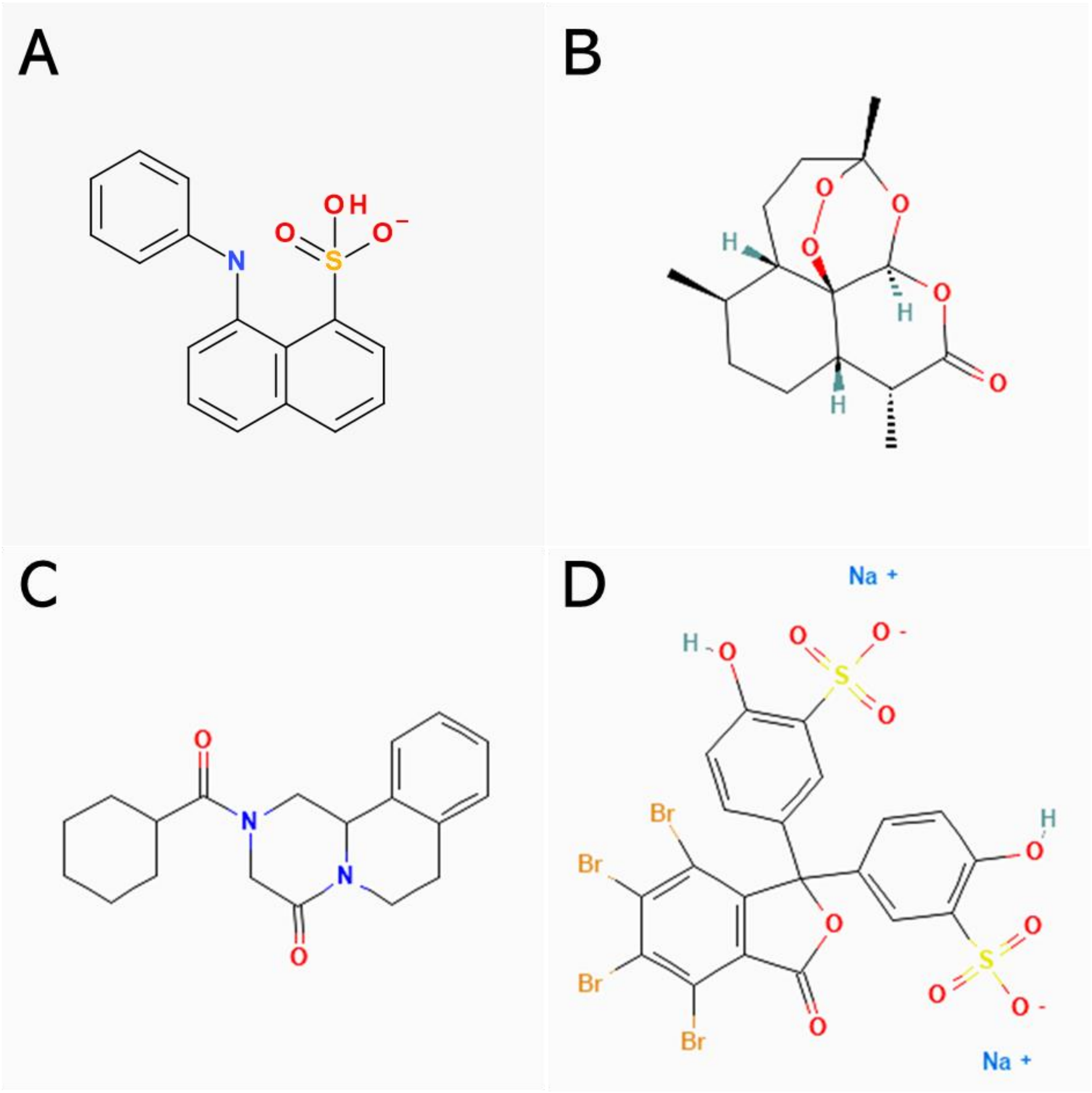

Figure S2: 2D illustration of the structures of the four ligands used in the study. ***A***. ANS, ***B***. Artemisinin, ***C***. Praziquantel and ***D***. Bromosulphthalein.

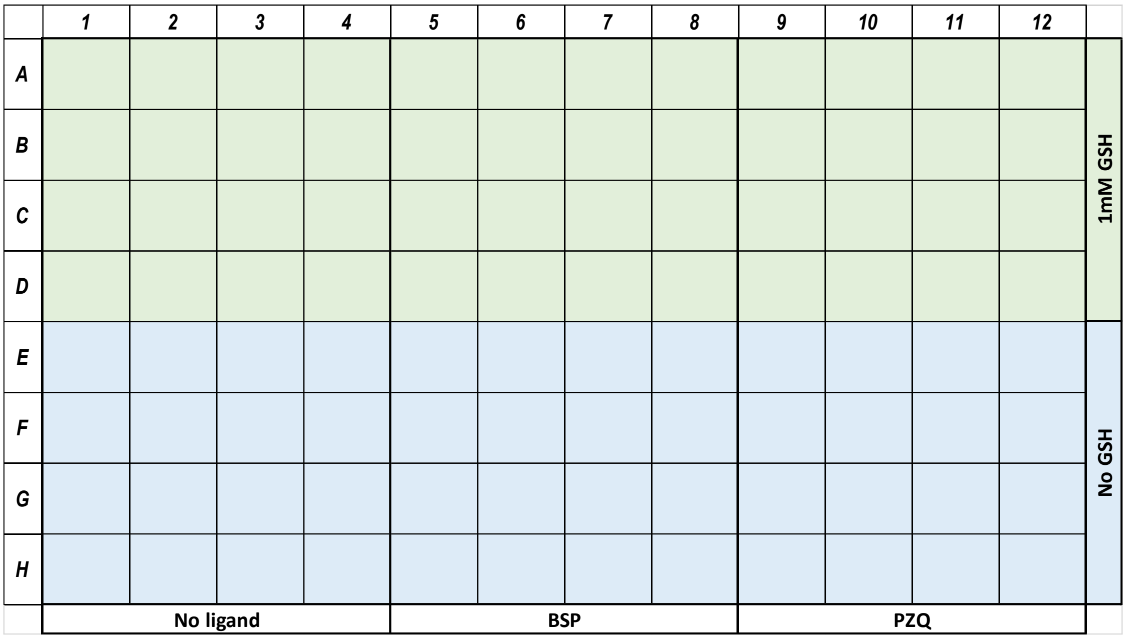

Figure S3: The 96-well plate plan used for the fluorescence-based thermal shift assay on the effect of PZQ and BSP on the stability of the pseudo Sbh26GST.

```
T-COFFEE, Version_11.00 (Version_11.00)
Cedric Notredame
SCORE=100
*
 BAD AVG GOOD
*
Sj26GST.     : 100
Sbh26GST.    : 100
cons         :  10

Sj26GST.     MSPILGYWKIKGLVQPTRLLLEYLEEKYEEHLYERDEGDKWRNKKFELGLEFPNLPYYIDGDVKLTQSM
Sbh26GST.    MAPILGYWKIKGLVQPTRLLLEYVGEVYEERLYDRNDGDVWRNEKFNLGLEFPNLPYYIDGDVKLTQSM

cons         *:*********************: * ***:**:*::** ***:**:***********************


Sj26GST.     AIIRYIADKHNMLGGCPKERAEISMLEGAVLDIRYGVSRIAYSKDFETLKVDFLSKLPEMLKMFEDRLC
Sbh26GST.    AILRYIADKHNMLGGCPKERAEISMLEGAILDIRLGVSRIAYNKEFETLKVGFLNQLPGMLKMFENRLS

cons         **:**************************:**** *******.*:******.**.:** ******:**.


Sj26GST.     HKTYLNGDHVTHPDFMLYDALDVVLYMDPMCLDAFPKLVCFKKRIEAIPQIDKYLKSSKYIAWPLQGWQ
Sbh26GST.    HKIYLNGDNVTHVDFMLYDALDVVLYMDPKCLDAFPKLISFKQRIENLPPIKNYLNSDRHIKWPLQGWS

cons         ** *****:*** **************** ********:.**:*** :* *.:**:*.::* ******.


Sj26GST.     ATFGGGDHPPK
Sbh26GST.    AIFGGGDAPPK

cons         * ***** ***
```

Figure S4. Computation of a 3-dimensional structural alignment between pseudo Sbh26GST and Sj26GST using the Expresso Alignment algorithm.

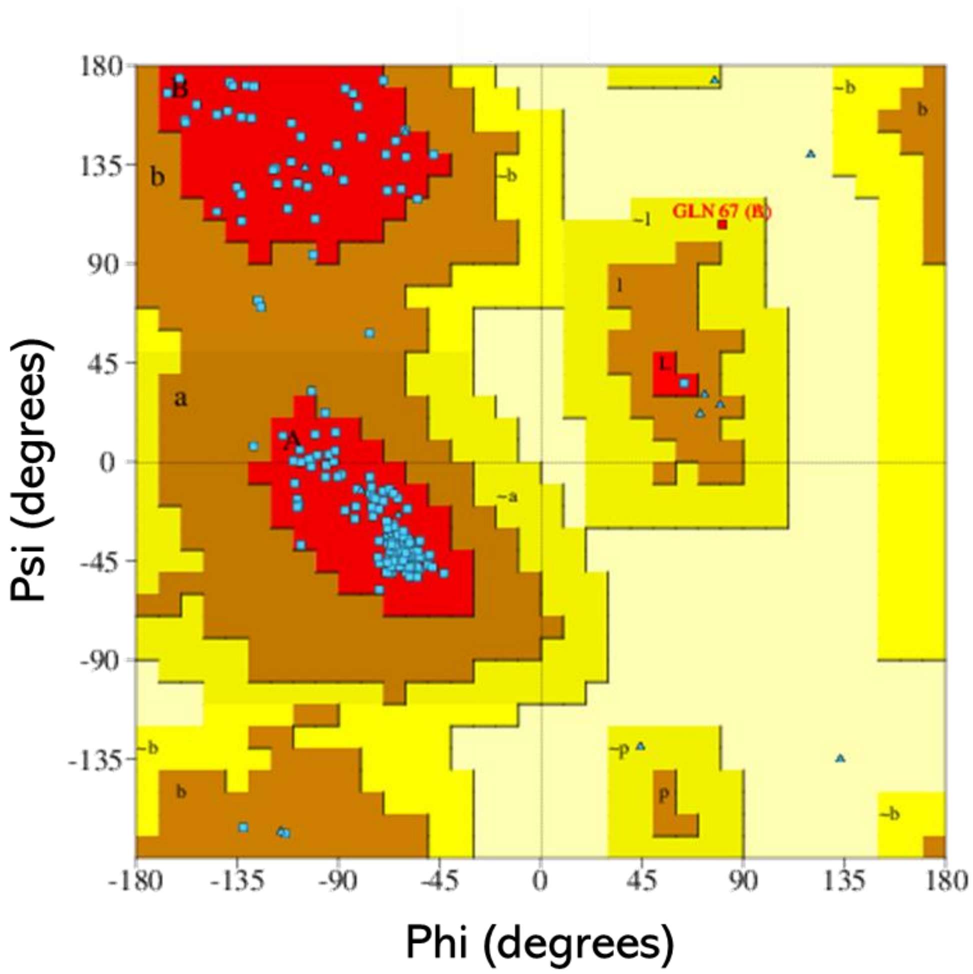

Figure S5. Ramachandran plot analysis of Sbh26GST. Here, the red region indicates favored region, yellow region for allowed and light-yellow shows generously allowed region and white for disallowed region. Phi and Psi angels define torsion angles is a figure. This analysis was done using the ProCheck algorithm [1, 2].

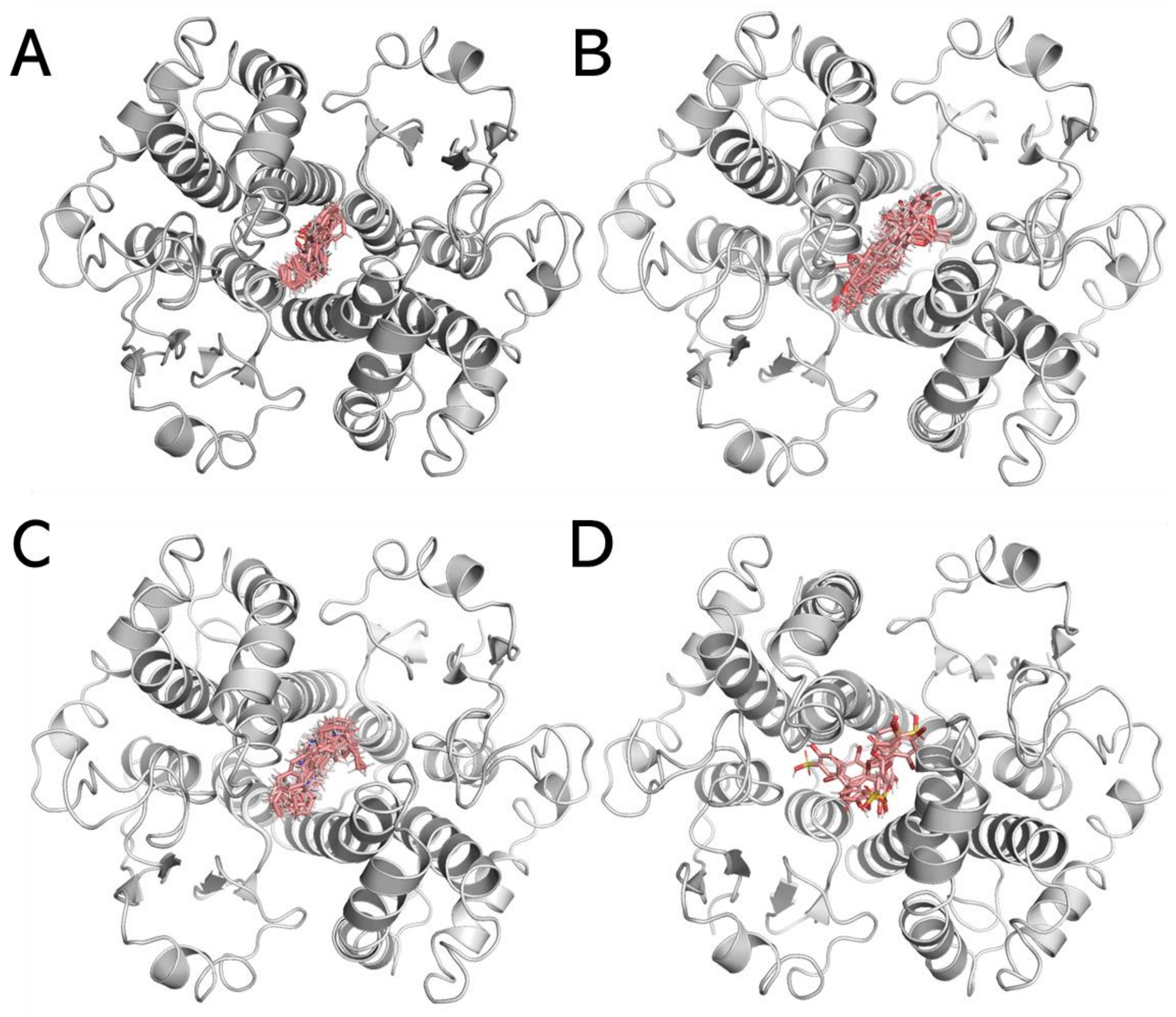

Figure S6. Ribbon representation of the 3D homology model of Sbh26GST indicating the preferred binding site for ANS (***A***), ART (***B***), PZQ (***C***) and BSP (***D***) based on the induced fit ligand docking implemented in Schrödinger Maestro v12.0. All the poses derived indicate that the ligands (in pink stick representation) preferred to bind at the dimer interface. The image was rendered using PyMol [3].

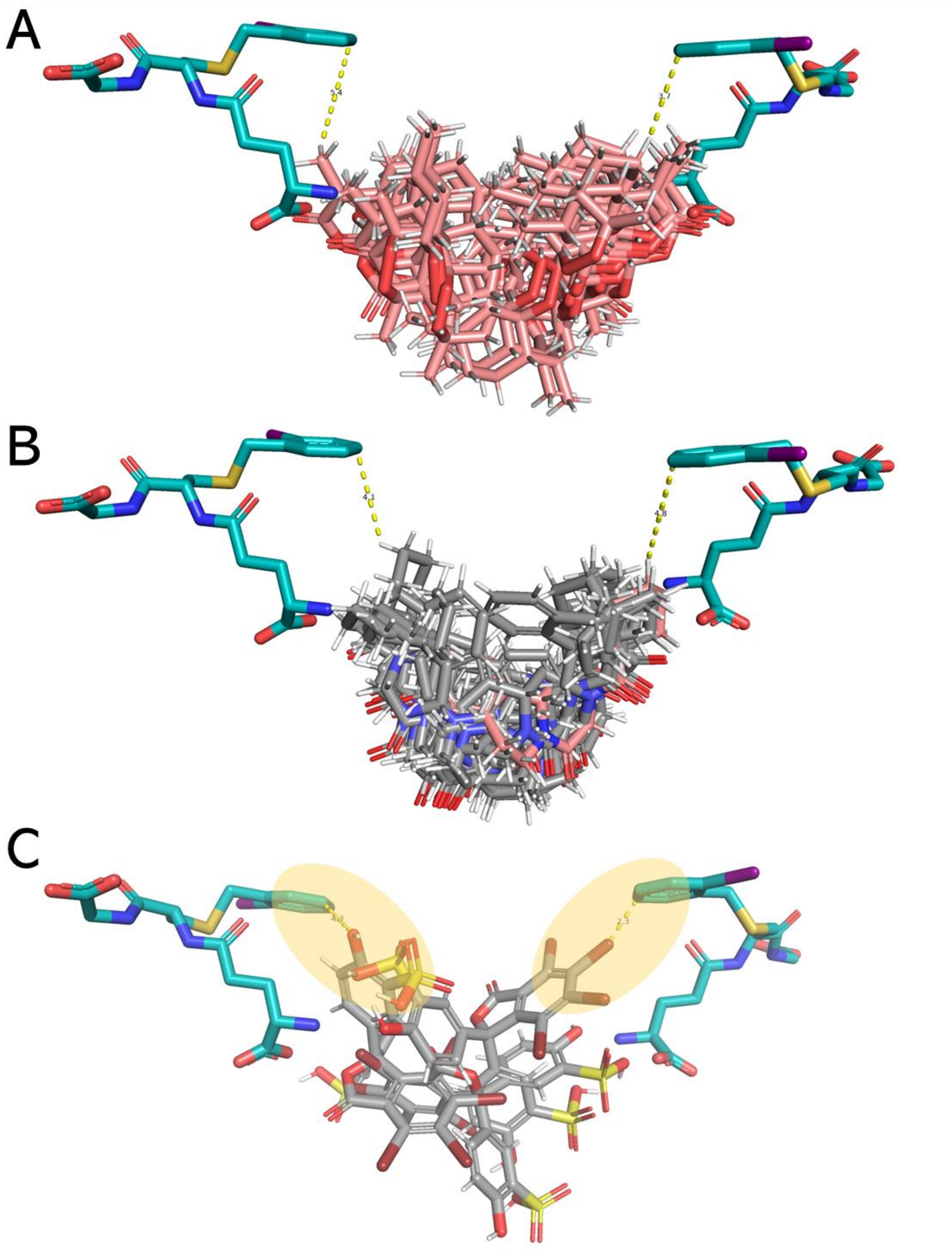

Figure S7. Stick representation of the proximity between the ligands bond within the dimer interface and the active sites occupied by the glutathione moiety in the G-site and the 2-iodobenzyl moiety occupying the H-site. ***A***. ART, ***B***. PZQ and ***C***. BSP. The image was generated using PyMol.

References

1. Laskowski, R.A.; MacArthur, M.W.; Moss, D.S.; Thornton, J.M. PROCHECK: a program to check the stereochemical quality of protein structures. *J Appl Crystallogr* **1993**, *26*, 283-91.
2. Laskowski, R.A.; Rullmann, J.A.C.; MacArthur, M.W.; Kaptein, R.; Thornton, J.M. AQUA and PROCHECK-NMR: programs for checking the quality of protein structures solved by NMR. *J Biomol NMR* **1996**, *8*, 477-86.
3. DeLano, W.L. Pymol: An open-source molecular graphics tool. *CCP4 Newsletter on protein crystallography* **2002**, *40*, 82-92.
